# A novel sternoclavicular hook plate for treatment of proximal clavicle fracture with dislocation of sternoclavicular join

**DOI:** 10.1186/s12893-022-01703-y

**Published:** 2022-06-29

**Authors:** Yadi Zhang, Baorui Xing, Xiuxiu Hou, Yunmei Li

**Affiliations:** Cangzhou Hospital of Integrated Traditional Chinese Medicine and Western Medicine, No. 31, West Huanghe Road, Yunhe, Cangzhou, 061000 Hebei China

**Keywords:** Sternoclavicular hook plate, Proximal clavicle fracture, Dislocation of sternoclavicular joint, Surgery

## Abstract

**Purpose:**

The aim of this study was to explore the efficacy of sternoclavicular hook plate for treatment of proximal clavicle fracture with dislocation of sternoclavicular joint.

**Methods:**

Between October 2016 and December 2020, 16 cases (9 male and 7 female patient, with a mean age of (42 ± 10) years) of proximal clavicle fracture with dislocation of sternoclavicular joint were included in the study. Injured side, injury mechanism, time to surgery, Complications and duration of follow-up were recorded. The outcomes were evaluated with radiographic assessment, American Shoulder and Elbow Surgeons’ Form (ASES). All the patients were evaluated on postoperative 3rd, 6th, and 12th months.

**Results:**

According to the ASES scoring system, the average score was 49 ± 4 (preoperative score), 87 ± 5 (3 months follow-up), 88 ± 3 (6 months follow-up) and 91 ± 3 (12 months follow-up). Statistics differences was showed between preoperative and 3,6,12 months follow-up score of ASES score (*p* < 0.001). The postoperative physical function was better than the preoperative function. Internal fixation failure and fracture non-union complications in two patients.

**Conclusion:**

Our study indicates that open reduction and sternoclavicular hook plate fixation for the treatment of traumatic sternoclavicular fracture is a safe, relatively straightforward surgical procedure that can lead to satisfactory outcomes.

## Introduction

The sternoclavicular joint can move freely in many directions, it allow for upward elevation, anteroposterior translation, and rotation along its long axis and contributes to scapulothoracic motion [1]. It’s the only synovial joint connecting the upper extremity and the trunk. The stability of the joint depends exclusively on the meniscus, joint capsule and the surrounding ligaments [2]. Proximal clavicle fracture is a rare injury. The incidence of proximal clavicle fractures have lower incidence than fracture of the middle third and distal clavicle fracture, accounting for 5–6% of the clavicle fractures [3, 4]. However, proximal clavicle fractures and sternoclavicular joint dislocation are not routinely divided into different injuries, which is a significant limitation of most research to date and therefore diluting the applicability of suggested treatment algorithms [5, 6]. Proximal clavicle fracture with sternoclavicular dislocation is a relatively rare fracture dislocation in clinical, often caused by violence. SCJ dislocation was presumably frequently missed, both clinically and radiographically. Kusnezov et al. report in their study group a rate of late diagnosis of more than 1 year in 71% of all cases [7]. With the widespread availability of CT scanning, it’s now straight-forward to diagnose yet it is still frequently missed.

Historically, a number of reconstruction techniques have been described. These all generally involve Kirschner wires [8], Steinmann pins [9], soft tissue reconstruction(suture, autologous tendon, allograft) [10, 11], plate techniques [12], sternoclavicular temporary arthrodesis [13], sternoclavicular buttress plate and sternoclavicular hook plate [14].There is little literature describe on proximal clavicle fracture with dislocation of sternoclavicular joint, but no gold standard has been establish. We describe our preferred surgical approach for treatment of proximal clavicle fracture with dislocation of sternoclavicular joint. Furthermore patient outcome and follow-up results are reported.

## Methods

### General data

All 17 cases of proximal clavicle fracture with dislocation of sternoclavicular joint treated between October 2016 and December 2020 in Cangzhou Hospital of integrated traditional Chinese medicine and Western medicine. Inclusion criteria: (1) Age > 16 years old; (2) Fractures that could not be reduced by nonoperative treatment and type D fresh proximal clavicle fractures according to the Throckmorton classification [15]; (3) Fractures that were prone to recurrence during shoulder movement and cases with prominent skin lesions; (4) Patients without underlying diseases such as heart disease. Exclusion criteria: (1) Patients with brain injury or severe underlying chronic disease and, therefore, cannot tolerate the risk of surgery and anesthesia; (2) Pathological fractures; (3) Open fractures; (4) Combined vascular or neurological injuries. (5) The patient requested conservative treatment, even though closed reduction was pointless (Table 1). All procedures were performed by the senior author after obtaining informed consent from all patients. Sixteen cases of proximal clavicle fracture with dislocation of sternoclavicular joint were included in the study. One patient was excluded due to an open fracture. Informed written consent for publication of this report and accompanying figures was obtained from the patients. The hospital research ethics committee approved the study.

**Table 1:** Inclusion criteria and exclusion criteria

Inclusion criteria	Exclusion criteria
Age > 16 years old	(1) Patients with brain injury or severe underlying chronic disease and, therefore, cannot tolerate the risk of surgery and anesthesia
Fractures that could not be reduced by nonoperative treatment and type D fresh proximal clavicle fractures according to the Throckmorton classification	(2) Pathological fractures
Fractures that were prone to recurrence during shoulder movement and cases with prominent skin lesions	(3) Open fractures
Patients without underlying diseases such as heart disease	(4) Combined vascular or neurological injuries
	The patient requested conservative treatment, even though closed reduction was pointless

**Fig. 1:**
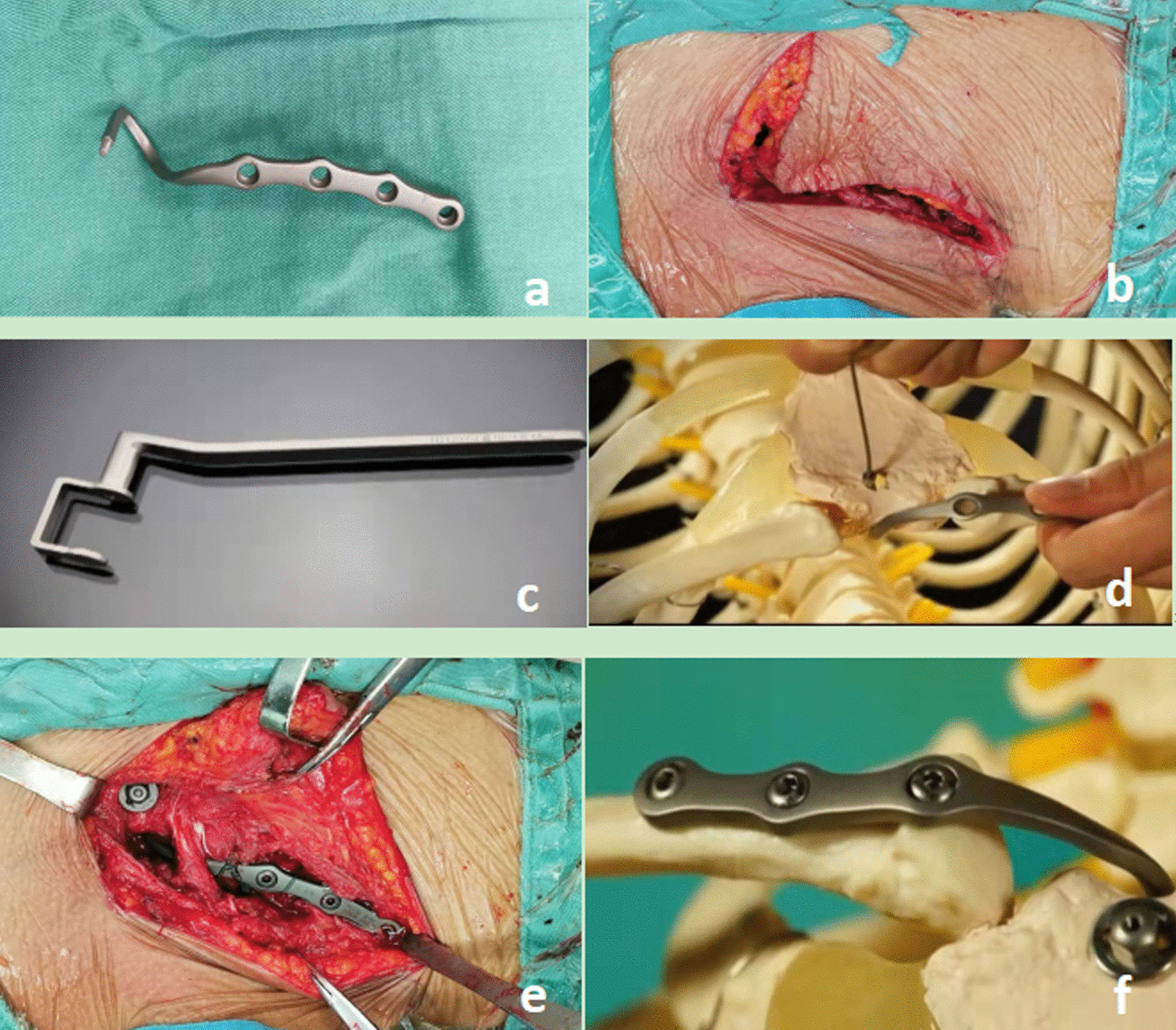
**a**, **b** Preoperative CT scan image show a right anterior dislocation of the sternoclavicular joint and proximal clavicle fracture, **c** Preoperative three-dimensional CT scan image, **d** Proximal end of the clavicle was displaced anteriorly and proximal clavicle fracture

Conventional radiographs are not sensitive for posterior sternoclavicular dislocations, and computed tomography (CT) represents the imaging modality of choice [16, 17]. All patients underwent the standard preoperative assessment, including preoperative history, physical examination, plain radiograph of the clavicle, sternum and thorax completed with serendipity view and a CT scan of the thorax (Fig. 1).

### Surgical technique

**Fig. 2:**
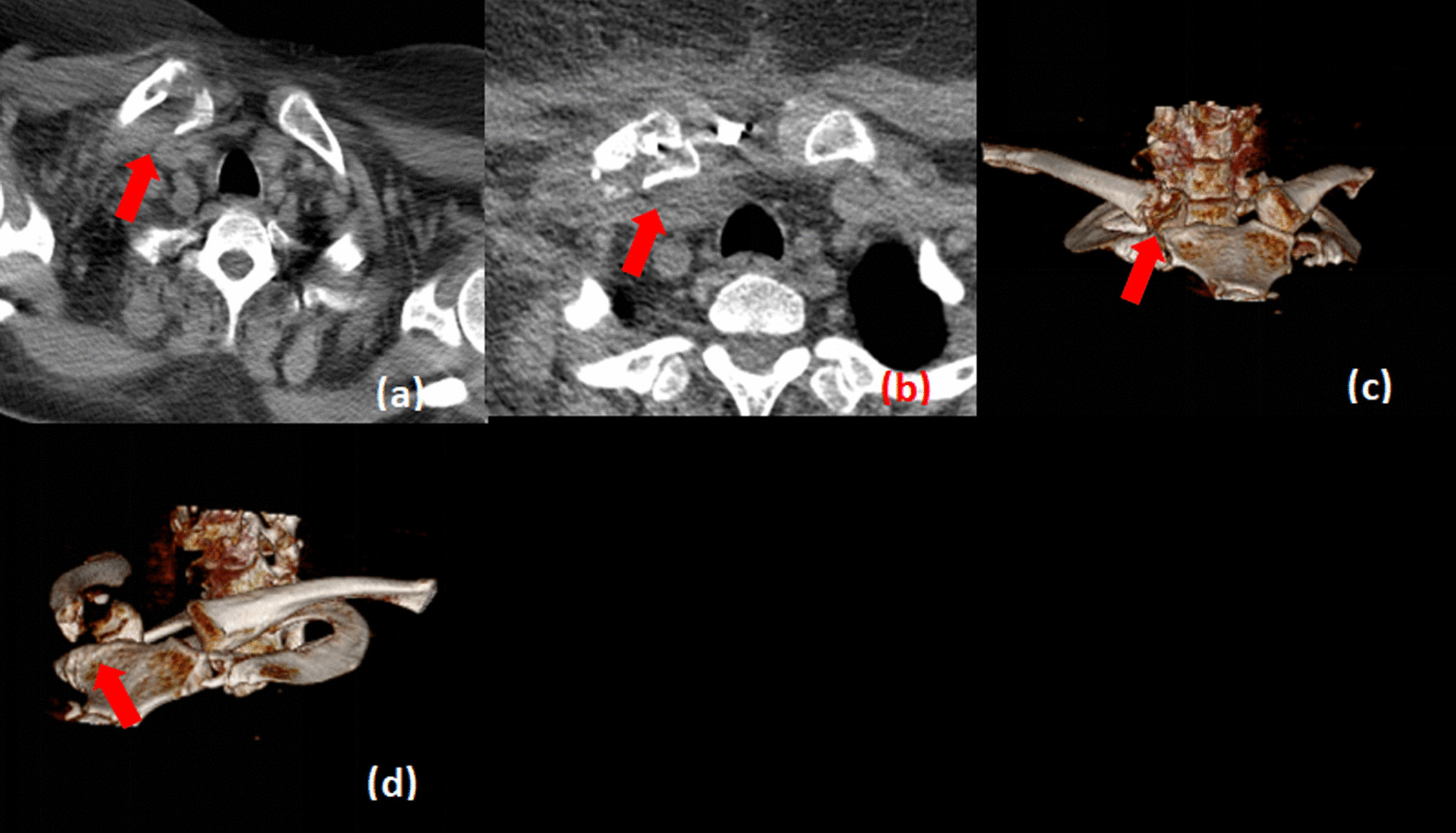
**a** Morphology of the plate; **b** anterior dislocation of the right sternoclavicular joint with an hockey-stick shaped incision; **c** "C" shaped protective sleeve; **d** The sternal stalk from posterior to anterior through the drilled hole; **e** The plate is fixed to the clavicle with screws to complete the fixation; **f** In case of posterior dislocation, the plate resets the fracture dislocation by lifting, and a screw spacer and screw cap are added to the threads at the hook end to prevent recurrence of posterior dislocation

All patients were positioned supine with free draping of the ipsilateral arm on the operating table, and underwent general anesthesia. Two surgeons perform surgery (years of experience > 5 years). An hockey-stick shaped incision of approximately 8–10 cm length was made from the medial clavicle (Fig. 2b). Exposure of the proximal clavicle and sternoclavicular joint. Protection of the lateral sternal termination point of the sternocleidomastoid muscle. Separate the appropriate tissue gap along the posterior aspect of the sternal stalk from the upper to the lower. A special "C" shaped protective sleeve (Fig. 2c) is placed through the sternal stalk and the sternal aperture is prepared by drilling a hole from front to back. A suitable sternoclavicular hook plate (CANWELL MEDICAL CO., LTD) (Fig. 2a) is selected and wound from the upper edge of the sternal stalk from posterior to anterior through the drilled hole (Fig. 2d). In anterior dislocation the clavicle is repositioned posteriorly using leverage, the proximal fracture is also repositioned, and then the plate is fixed to the clavicle with screws to complete the fixation (Fig. 2e). In case of posterior dislocation, the plate resets the fracture dislocation by lifting, and a screw spacer and screw cap are added to the threads at the hook end to prevent recurrence of posterior dislocation (Fig. 2f). Intraoperatively, the proximal clavicle fracture and sternoclavicular joint dislocation were well repositioned and the internal fixation plate was well positioned under C-arm X-ray fluoroscopy. The incision was rinsed, and the incision was closed with sutures after careful hemostasis.

### Postoperative management

**Fig. 3:**
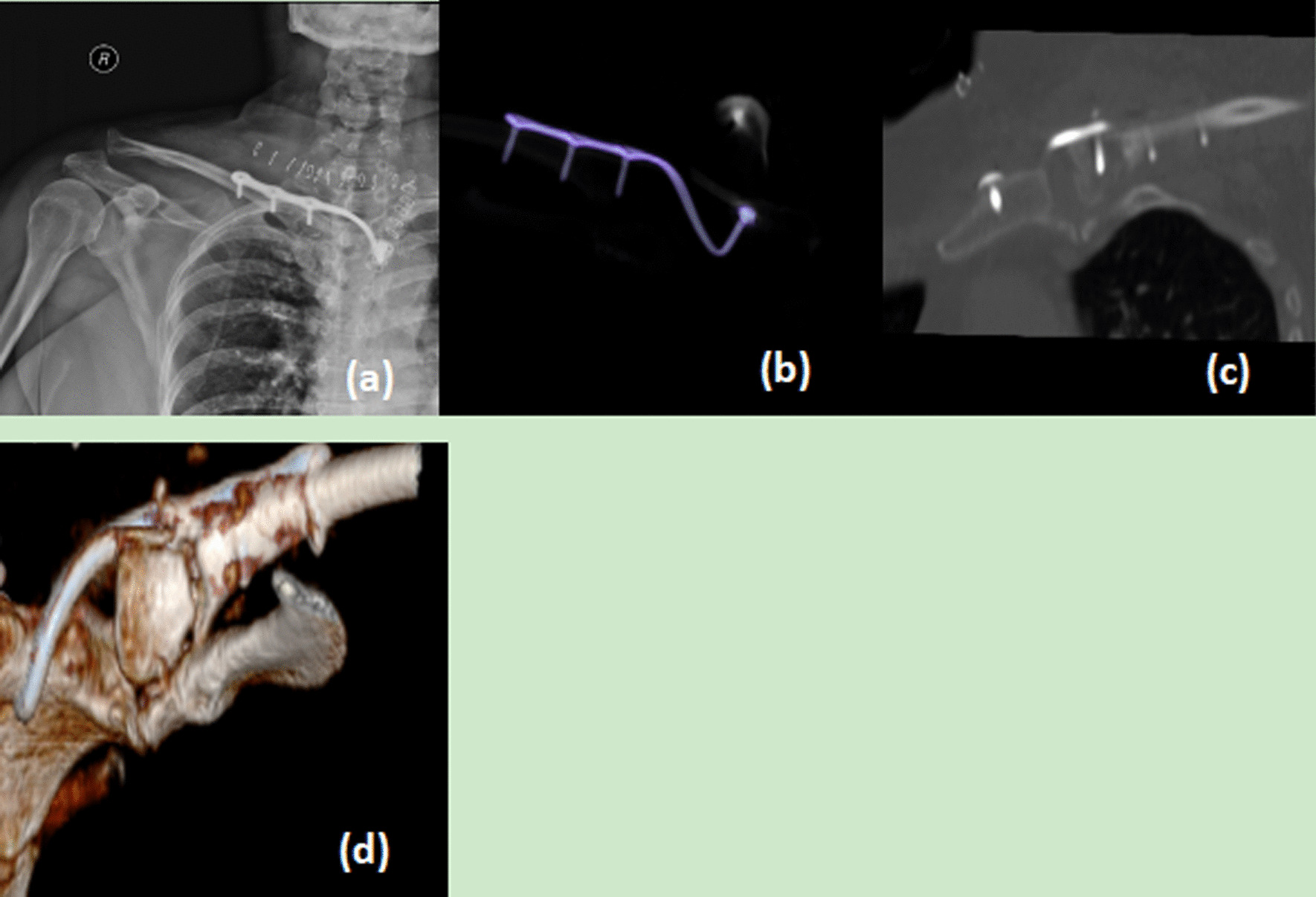
**a**, **b** The postoperative X-ray image showed the well reduction by CanHSH sternoclavicular hook plate, **c**, **d** postoperative CT scan and three-dimensional CT scan of the patient after 3 months follow up

Three days after surgery, patients were allowed to do passive forward flexion and abduction of the shoulder joint under the guidance of the physical therapist. Depending on the degree of pain and postoperative X-ray and CT scan images, the range of motion could be gradually increased, and no weight-bearing exercise was allowed. Weight-bearing exercises were gradually started after 6 weeks postoperatively. Postoperative follow-up is every 4 weeks until the bone heals, and every 3 months after the bone heals, and exercise should be avoided for 12 weeks after surgery. The sternoclavicular plate can be removed at 12 months after surgery according to the clinical course (Fig. 3).

### Outcome measures

**Fig. 4:**
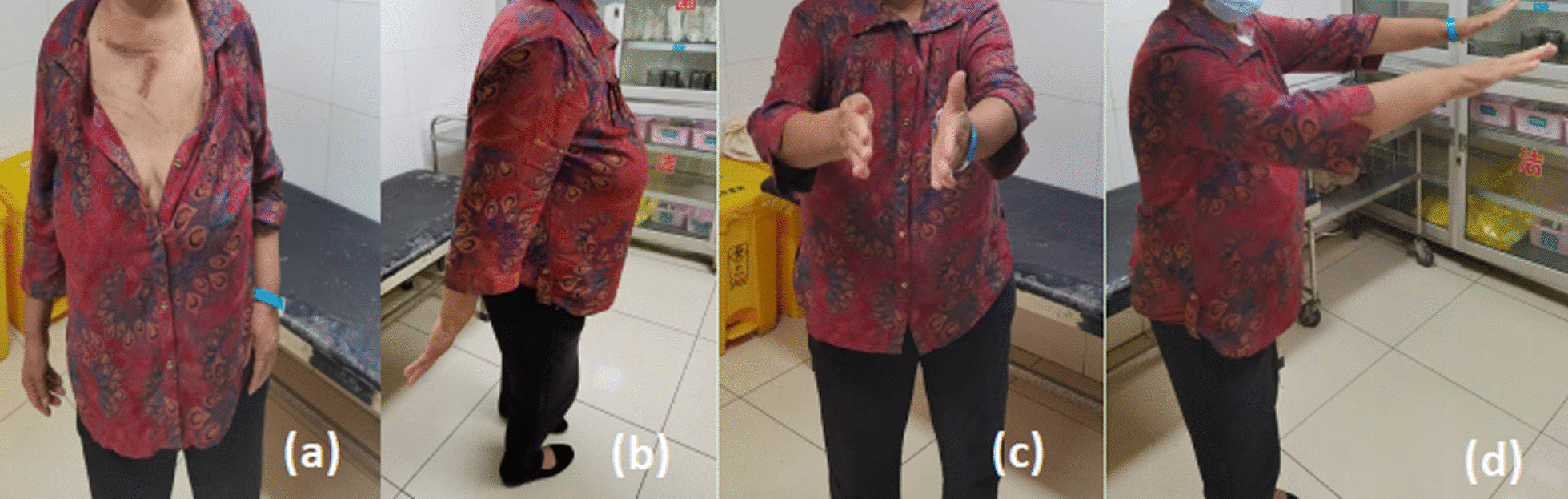
**a** Resting position；** b** Dorsal extension position；** c**,** d** Front lifting position. Functional outcome 3 months after reconstruction of anterior sternoclavicular joint dislocation and proximal clavicle fracture

Three raters assessed the clinical outcome by American Shoulder and Elbow Surgeons’ Form (ASES) [18]. The system is a 100-point system consisting of the patient's own assessment section (50%) and the cumulative daily activities section (50%). Patients were assessed for pain, stability, and daily activities. Part of the doctor's assessment is mobility, signs, strength tests, and stability. The higher the score, the better shoulder function. VAS was used to evaluate the pain scale [19]. The life function scale included 10 daily activities: dressing, combing hair, and going to the toilet. Placzek et al. [20] found that ASES score had low correlation with age and high credibility through studies. Patients were routinely followed at 2 weeks and 9–12 months postoperatively. No significant complications occurred during follow-up (Fig. 4).

Fracture healing standards [21]: (1) There was no local pressure pain and no longitudinal percussion pain. (2) No abnormal local activity. (3) X-ray shows blurred fracture line with continuous bone scab through the fracture line. Fracture non-union standards [22]: (1) The fracture did not heal 6 to 9 months after the fracture. (2) Dynamic observation of the fracture site for 3 months with no signs of healing. (3) There was pressure pain and longitudinal percussion pain at the fracture site. (4) X-ray suggested sparse bone scabs at the fracture site, sclerosis at the fracture site, and closure of the medullary cavity.

### Statistical analysis

All statistical data were statistically analyzed using the statistical software SPSS 20.0 (Statistical Package for Social Sciences, SPSS Inc, Chicago, IL, USA) to calculate the results of each measure, and the values are expressed as mean ± standard deviation. Count data were analyzed by student’s t-test, and P < 0.05 was set as a statistically significant difference.

## Results

A power analysis was performed. T tests—Means: Difference from constant (one sample case). Analysis: A priori: Compute required sample size. Input: Tail(s) = Two; Effect size d = 0.8; α err prob = 0.05; Power (1-β err prob) = 0.8. Output: Noncentrality parameter δ = 3.0983867; Critical t = 2.1447867; Df = 14; Total sample size = 15; Actual power = 0.8213105. There were 9 male patients and 7 female patients with a mean age of (42 ± 10) years. Four patients had unilateral dislocation, 12 patients had proximal clavicle fractures, and 1 of the 16 patients had old dislocation (more than 3 weeks). One patient had a rib fracture; mechanism of injury: 12 patients were in a car injury and 4 patients were injured by an impact object. Age, gender distribution, side involved, cause of injury, type of dislocation, associated injury, time between injury and surgery, and follow-up are shown in Table 2. According to the ASES scoring system, the average score was 49 ± 4 (preoperative score), 87 ± 5 (3 months follow-up), 88 ± 3 (6 months follow-up) and 91 ± 3 (12 months follow-up). Statistics differences was showed between preoperative and 3, 6, 12 months follow-up score of ASES score (*p* < 0.001). The postoperative physical function was better than the preoperative function (Table 3). Internal fixation failure and fracture non-union complications in 2 patients (Table3).

**Table 2:** Patient characteristics

Patient	Gender	Age (years)	Side	Injury mechanism	Time to surgery	Follow-up (months)
1	F	49	L	Road traffic accident	1 week	14
2	M	37	L	Bike accident	2 days	12
3	M	52	R	Bike accident	1 week	18
4	F	48	L	Crashing object	3 days	16
5	F	41	R	Road traffic accident	5 days	21
6	M	25	R	Road traffic accident	4 weeks	23
7	M	56	L	Crashing object	5 days	13
8	F	34	R	Crashing object	3 days	13
9	F	42	L	Road traffic accident	6 days	12
10	M	44	R	Bike accident	3 days	14
11	F	28	R	Road traffic accident	1 week	13
12	M	27	L	Bike accident	3 days	15
13	M	56	R	Crashing object	5 days	18
14	M	53	L	Road traffic accident	1 week	21
15	F	48	R	Bike accident	1 day	13
16	M	36	L	Bike accident	5 days	13

**Table 3:** Preoperative, 3-, 6-, and 12-month postoperative follow-up of ASES (American Shoulder and Elbow Surgeons’ Form) and complications of proximal clavicle fracture with dislocation of sternoclavicular joint

Patient	ASES Score (pre-op)	ASES Score (3 months post-op)	ASES Score (6 months post-op)	ASES Score (12 months post-op)	Complications
1	46	80	86	93	None
2	53	86	90	90	None
3	48	93	93	93	None
4	60	83	85	85	Failed internal fixation and displaced fracture
5	45	92	93	93	None
6	46	80	85	90	None
7	50	90	90	90	None
8	52	85	86	92	None
9	47	85	92	90	None
10	52	93	90	95	None
11	49	84	86	95	None
12	48	92	91	92	None
13	48	93	86	85	Fracture non-union
14	46	83	85	93	None
15	50	92	85	90	None
16	52	80	90	90	None
Mean ± s	49 ± 4	87 ± 5	88 ± 3	91 ± 3	
p		< 0.001	< 0.001	< 0.001	

## Discussion

The sternoclavicular hook plate is a special type of plate designed for the treatment of sternoclavicular dislocation, which is applied to treat proximal clavicle fracture with sternoclavicular dislocation. Zhang et al. have briefly introduced sternoclavicular hook plate, it can provide micromovement that facilitates shoulder girdle movement [14]. In this study, an sternoclavicular joint hook plate was used for the treatment of proximal clavicle fracture with dislocation of sternoclavicular join. There are some advantages of the treatment using the sternoclavicular hook plate: (1) The anterior medial side of the clavicle is fixed with cortical screws, which not only provides stability, but also allows the hook plate to provide a certain range of fretting when the sternoclavicular joint is active; (2) Patients can exercise shoulder joint in the early stage after surgery; (3) Sternoclavicular dislocation with proximal clavicular fracture can be fixed to provide sufficient strength to maintain proximal clavicular fracture; (4) The hook end of the plate passes behind the sternocleidomastoid muscle, crosses the upper margin of the manubrium sternum and penetrates from the manubrium sternum body, avoiding the risk of intraoperative damage to important blood vessels and lungs.

In our report, we performed incisional repositioning sternoclavicular hook plate fixation in 16 patients with proximal clavicle fracture with dislocation of sternoclavicular joint. Postoperatively, good shoulder motion was obtained with no discomfort at the 2-month postoperative follow-up. One patient presented with pain at the sternoclavicular joint where the plate was placed at the 1-month postoperative review. X-ray examination suggested that the fracture was displaced by internal fixation failure. The patient was engaged in more active work of the upper extremity. We removed the sternoclavicular plate. The patient was treated conservatively.

The SCJ is a relatively unstable saddle synovial joint. Based on its anatomy, we can understand that the stability of the SCJ is maintained by its periarticular ligaments. Also the activity of the shoulder joint complex can cause the movement of the SCJ [23]. When the arm is raised 120°, the clavicular end of the sternoclavicular joint is posteriorly rotated 15°, raised 5°, and internally retracted 15°. The stability of the SCJ is maintained mainly by the posterior sternoclavicular ligament, whose rupture will lead to joint instability and posterior dislocation [24].

There is still no consensus regarding the treatment of proximal clavicle fracture with dislocation of sternoclavicular joint. Groh et al. report on a trial of closed reduction in 21 patients, which was effective in only eight patients [25]. Previous studies have also shown that closed reduction of sternoclavicular dislocation has a high risk of redislocation [26]. Even after successful closed reduction, residual sternoclavicular instability still exists, requiring subsequent secondary surgery [27]. If closed reduction does not work, then open treatment should be considered. Various techniques have been proposed for the surgical management of proximal clavicle fracture with dislocation of sternoclavicular joint. Considering the structural characteristics of the SCJ, the application of k-wire fixation will lead to the displacement of the k-wire due to the activity of the SCJ, which in turn will lead to the injury of the surrounding large vessels. The application of locking plate fixation of proximal clavicle fracture with dislocation of sternoclavicular joint will result in restricted movement of the joint and loss of most of its function. With increasing attention to the degree of postoperative pain and functional recovery, locking plate fixation of proximal clavicle fracture with dislocation of sternoclavicular joint has gradually been discouraged for this joint [28]. The transfer of the sternocleidomastoid tendon or the subclavius tendon has been described [29]. The failure rate of tendon autografting may be related to the complexity of the technique, operation time and the site where the tendon is obtained [29, 30].

There are some limitations in this study: Proximal clavicle fracture with dislocation of sternoclavicular joint is a rare lesion, the sample size of this study is small, the occurrence of type II error cannot be completely avoided. The further expansion of the sample size in multicenter studies is needed to support our findings. Also, we did not have a randomized control group, and the specific advantages and disadvantages of this procedure over other procedures need to be further investigated.

## Conclusion

Our study shows that adopting open reduction and sternoclavicular hook plate fixation for traumatic sternoclavicular dislocation or fracture is a safe and relatively simple procedure that can achieve satisfactory results.

## Data Availability

The patients’ dataset are confidential and are privately held for patients confidentiality safeguard. As such, the datasets generated and/or analysed during the current study are not publicly available but are available from the corresponding author on reasonable request.
